# Supplementary material for: Efficient Algorithms and Implementation of a Semiparametric Joint Model for Longitudinal and Competing Risk Data: With Applications to Massive Biobank Data
Source: Comput Math Methods Med. 2022 Feb 8;2022:1362913. doi: 10.1155/2022/1362913 (PMC8846996; doi:10.1155/2022/1362913)
Supplement: Supplementary Materials — Derivations of formulas, additional simulation results, and analysis results of the two real data are provided in the supplementary materials. [file 1362913.f1.zip › supp (2).pdf]

APPENDIX A. Supplementary Materials for "Efficient Algorithms and Implementation of a Semiparametric Joint Model for Longitudinal and Competing Risks Data: With Applications to Massive Biobank Data"

APPENDIX A.1. M-step solutions for equation (4) in Section 2.1.2

It can be shown that the parameters β, σ^2, and Σ, as well as $\Lambda_{0k}(t)$ have closed-form solutions in the M-step (See equation (4)). Using $E^{(m)}$ to denote $E^{(m)}_{b_i|Y_i,C_i,\Psi^{(m)}}$, we have

$$\begin{aligned}
\beta^{(m+1)} &= \left\{\sum_{i=1}^{n}\sum_{j=1}^{n_i} X_i^{(1)}(t_{ij})X_i^{(1)}(t_{ij})^T\right\}^{-1}\sum_{i=1}^{n}\sum_{j=1}^{n_i}\left\{Y_{ij}-E^{(m)}(\tilde{X}_i^{(1)}(t_{ij})^T b_i)\right\} \\
&\quad \times X_i^{(1)}(t_{ij}), \qquad (A.1)\\
\sigma^{2(m+1)} &= \frac{1}{\sum_{i=1}^{n} n_i}\sum_{i=1}^{n}\sum_{j=1}^{n_i} E^{(m)}\left\{Y_{ij}-X_i^{(1)}(t_{ij})^T\beta^{(m+1)}-\tilde{X}_i^{(1)}(t_{ij})^T b_i\right\}^2, \qquad (A.2)\\
\Sigma^{(m+1)} &= \frac{1}{n}\sum_{i=1}^{n} E^{(m)}(b_i b_i^T), \qquad (A.3)\\
\Lambda_{0k}^{(m+1)}(t) &= \sum_{l:t_{kl}\le t}\frac{d_{kl}}{\sum_{r\in R(t_{kl})}\exp(X_r^{(2)T}\gamma_k^{(m)})E^{(m)}\left\{\exp(\nu_k^{(m)T} b_r)\right\}}, \qquad (A.4)
\end{aligned}$$

where $t_{k1} > ... > t_{kq_k}$ are the distinct uncensored failure times for risk k, $R(t_{kl})$ is the risk set at time t_{kl}, $l = 1, ..., q_k$, and d_{kl} is the number of type k failures, for $k = 1, \ldots, K$. It is clear from equation (A.4) that $\Lambda_{0k}^{(m+1)}(t)$ is a right-continuous and non-decreasing function.

The parameters γ and ν do not have closed-form solutions and we update them using the one-step Newton-Raphson method

$$\begin{aligned}
\gamma_k^{(m+1)} &= \gamma_k^{(m)} + I_{\gamma_k}^{(m)-1} S_{\gamma_k}^{(m)}, \quad k = 1, \ldots, K,\\
\nu_k^{(m+1)} &= \nu_k^{(m)} + I_{\nu_k}^{(m)-1} S_{\nu_k}^{(m)}, \quad k = 1, \ldots, K,
\end{aligned}$$

where

$$
\begin{aligned}
I_{\gamma_k}^{(m)} &= \sum_{i=1}^{n} \sum_{t_{kj} \le T_i} \Delta\Lambda_{0k}(t_{kj})^{(m+1)} \exp(X_i^{(2)T} \gamma_k^{(m)}) E^{(m)} \left\{ \exp(\nu_k^{(m)T} b_i) \right\} X_i^{(2)} X_i^{(2)T}, \quad \text{(A.5)} \\
S_{\gamma_k}^{(m)} &= \sum_{i=1}^{n} \Big[I(D_i = k) X_i^{(2)}(T_i) \\
&\quad - \sum_{t_{kj} \le T_i} \Delta\Lambda_{0k}(t_{kj})^{(m+1)} \exp(X_i^{(2)T} \gamma_k^{(m)}) E^{(m)} \left\{ \exp(\nu_k^{(m)T} b_i) \right\} X_i^{(2)} \Big], \quad \text{(A.6)} \\
I_{\nu_k}^{(m)} &= \sum_{i=1}^{n} \sum_{t_{kj} \le T_i} \Delta\Lambda_{0k}^{(m+1)} \exp(X_i^{(2)T} \gamma_k^{(m)}) E^{(m)} \left\{ b_i b_i^T \exp(\nu_k^{(m)T} b_i) \right\}, \quad \text{(A.7)} \\
S_{\nu_k}^{(m)} &= \sum_{i=1}^{n} \Big[I(D_i = k) E^{(m)}(b_i) \\
&\quad - \sum_{t_{kj} \le T_i} \Delta\Lambda_{0k}(t_{kj})^{(m+1)} \exp(X_i^{(2)T} \gamma_k^{(m)}) E^{(m)} \left\{ b_i \exp(\nu_k^{(m)T} b_i) \right\} \Big]. \quad \text{(A.8)}
\end{aligned}
$$

APPENDIX A.2. Derivations of equation (11) in Section 2.2.1

Equation (11) in Section 2.2.1 can be derived as follows:

$$
\begin{aligned}
& E_{b_i|Y_i,C_i,\Psi^{(m)}}^{(m)} \{h(b_i)\} \\
= & \int h(b_i) f(b_i|Y_i, C_i, \Psi^{(m)}) db_i \\
= & 2^{\frac{q}{2}} |\tilde{H}_i|^{-1/2} \int h(\tilde{b_i} + \sqrt{2}\tilde{H}_i^{-1/2} c_i) \frac{f(\tilde{b_i} + \sqrt{2}\tilde{H}_i^{-1/2} c_i | Y_i, C_i, \Psi^{(m)})}{\exp\{-||c_i||^2\}} \exp\{-||c_i||^2\} dc_i \\
\approx & 2^{\frac{q}{2}} |\tilde{H}_i|^{-1/2} \sum_{t_1,t_2,\ldots,t_q} \pi_t h(\tilde{b_i} + \sqrt{2}\tilde{H}_i^{-1/2} c_t) f(\tilde{b_i} + \sqrt{2}\tilde{H}_i^{-1/2} c_t | Y_i, C_i, \Psi^{(m)}) \exp\{||c_t||^2\} \\
= & 2^{\frac{q}{2}} |\tilde{H}_i|^{-1/2} \sum_{t_1,t_2,\ldots,t_q} \pi_t h(\tilde{r}_t) f(\tilde{r}_t | Y_i, C_i, \Psi^{(m)}) \exp\{||c_t||^2\} \\
= & 2^{\frac{q}{2}} |\tilde{H}_i|^{-1/2} \sum_{t_1,t_2,\ldots,t_q} \pi_t h(\tilde{r}_t) \frac{f(Y_i, C_i | \tilde{r}_t, \Psi^{(m)}) f(\tilde{r}_t | \Psi^{(m)})}{f(Y_i, C_i | \Psi^{(m)})} \exp\{||c_t||^2\} \\
= & \frac{2^{\frac{q}{2}} |\tilde{H}_i|^{-1/2} \sum_{t_1,t_2,\ldots,t_q} \pi_t h(\tilde{r}_t) \frac{f(Y_i, C_i | \tilde{r}_t, \Psi^{(m)}) f(\tilde{r}_t | \Psi^{(m)})}{f(Y_i, C_i | \Psi^{(m)})} \exp\{||c_t||^2\}}{2^{\frac{q}{2}} |\tilde{H}_i|^{-1/2} \sum_{t_1,t_2,\ldots,t_q} \pi_t \frac{f(Y_i, C_i | \tilde{r}_t, \Psi^{(m)}) f(\tilde{r}_t | \Psi^{(m)})}{f(Y_i, C_i | \Psi^{(m)})} \exp\{||c_t||^2\}} \\
= & \frac{2^{\frac{q}{2}} |\tilde{H}_i|^{-1/2} \sum_{t_1,t_2,\ldots,t_q} \pi_t h(\tilde{r}_t) f(Y_i, C_i | \tilde{r}_t, \Psi^{(m)}) f(\tilde{r}_t | \Psi^{(m)}) \exp\{||c_t||^2\}}{2^{\frac{q}{2}} |\tilde{H}_i|^{-1/2} \sum_{t_1,t_2,\ldots,t_q} \pi_t f(Y_i, C_i | \tilde{r}_t, \Psi^{(m)}) f(\tilde{r}_t | \Psi^{(m)}) \exp\{||c_t||^2\}} \\
= & \frac{\sum_{t_1,t_2,\ldots,t_q} \pi_t h(\tilde{r}_t) f(Y_i, C_i | \tilde{r}_t, \Psi^{(m)}) f(\tilde{r}_t | \Psi^{(m)}) \exp\{||c_t||^2\}}{\sum_{t_1,t_2,\ldots,t_q} \pi_t f(Y_i, C_i | \tilde{r}_t, \Psi^{(m)}) f(\tilde{r}_t | \Psi^{(m)}) \exp\{||c_t||^2\}},
\end{aligned}
$$

where the second equality is obtained by letting $b_i = \tilde{b_i} + \sqrt{2}\tilde{H_i}^{-1/2} c_i$, and the next step is obtained by applying the standard Gauss Hermite quadrature rule.

APPENDIX A.3. The observed score vector in equation (6) in Section 2.1.3

The components of the observed score vector in equation (6) are defined by

$$\nabla_\beta l^{(i)}(\hat\Omega; Y, C) = \frac{1}{\sigma^2}\sum_{i=1}^{n_i} E\left\{Y_{ij} - X_i^{(1)}(t_{ij})^T\beta - \tilde{X}_i^{(1)}(t_{ij})^T b_i\right\} X_i^{(1)}(t_{ij})\Bigg|_{\beta=\hat\beta, \sigma^2=\hat\sigma^2}, \quad \text{(A.9)}$$

$$\nabla_\Sigma l^{(i)}(\hat\Omega; Y, C) = \frac{1}{2}\left[2\Sigma^{-1}E(b_i b_i^T)\Sigma^{-1} - \left\{\Sigma^{-1}E(b_i b_i^T)\Sigma^{-1} \circ I\right\} - 2\Sigma^{-1} + \Sigma^{-1} \circ I\right]\Bigg|_{\Sigma=\hat\Sigma}, \quad \text{(A.10)}$$

$$\nabla_{\sigma^2} l^{(i)}(\hat\Omega; Y, C) = \left[\frac{1}{2\sigma^4}\sum_{i=1}^{n_i} E\left\{Y_{ij} - X_i^{(1)}(t_{ij})^T\beta - \tilde{X}_i^{(1)}(t_{ij})^T b_i\right\}^2 - \frac{n_i}{2\sigma^2}\right]\Bigg|_{\beta=\hat\beta, \sigma^2=\hat\sigma^2}, \quad \text{(A.11)}$$

$$\begin{aligned}\nabla_{\gamma_k} l^{(i)}(\hat\Omega; Y, C) = {} & I(D_i = k)\left[X_i^{(2)} - \frac{\sum_{r\in R(T_i)}\exp(\gamma_k^T X_r^{(2)})E\left\{\exp(\nu_k^T b_r)\right\}X_r^{(2)}}{\sum_{r\in R(T_i)}\exp(\gamma_k^T X_r^{(2)})E\left\{\exp(\nu_k^T b_r)\right\}}\right] \\ & + \left(\sum_{j:t_{kj}\le T_i}\frac{d_{kj}\sum_{r\in R(t_{kj})}\exp(\gamma_k^T X_r^{(2)})E\left\{\exp(\nu_k^T b_r)\right\}X_r^{(2)}}{\left[\sum_{r\in R(t_{kj})}\exp(\gamma_k^T X_r^{(2)})E\left\{\exp(\nu_k^T b_r)\right\}\right]^2}\right. \\ & \left. - \sum_{j:t_{kj}\le T_i}\frac{d_{kj}}{\sum_{r\in R(t_{kj})}\exp(\gamma_k^T X_r^{(2)})E\left\{\exp(\nu_k^T b_r)\right\}}X_i^{(2)}\right) \\ & \times \exp(\gamma_k^T X_i^{(2)})E\left\{\exp(\nu_k^T b_i)\right\}\Bigg|_{\gamma_k=\hat\gamma_k, \nu_k=\hat\nu_k}, \end{aligned} \quad \text{(A.12)}$$

$$\begin{aligned}\nabla_{\nu_k} l^{(i)}(\hat\Omega; Y, C) = {} & I(D_i = k)\left[E(b_i) - \frac{\sum_{r\in R(T_i)}\exp(\gamma_k^T X_r^{(2)})E\left\{b_r\exp(\nu_k^T b_r)\right\}}{\sum_{r\in R(T_i)}\exp(\gamma_k^T X_r^{(2)})E\left\{\exp(\nu_k^T b_r)\right\}}\right] \\ & + \left(\sum_{j:t_{kj}\le T_i}\frac{d_{kj}\sum_{r\in R(t_{kj})}\exp(\gamma_k^T X_r^{(2)})E\left\{b_r\exp(\nu_k^T b_r)\right\}}{\left[\sum_{r\in R(t_{kj})}\exp(\gamma_k^T X_r^{(2)})E\left\{\exp(\nu_k^T b_r)\right\}\right]^2}E\left\{\exp(\nu_k^T b_i)\right\}\right. \\ & \left. - \sum_{j:t_{kj}\le T_i}\frac{d_{kj}}{\sum_{r\in R(t_{kj})}\exp(\gamma_k^T X_r^{(2)})E\left\{\exp(\nu_k^T b_r)\right\}}E\left\{b_i\exp(\nu_k^T b_i)\right\}\right) \\ & \times \exp(\gamma_k^T X_i^{(2)})\Bigg|_{\gamma_k=\hat\gamma_k, \nu_k=\hat\nu_k}. \end{aligned} \quad \text{(A.13)}$$

APPENDIX A.4. Comparison of estimation results using the standard Gauss-Hermite quadrature rule and the pseudo-adaptive Gauss-Hermite quadrature rule

We ran a small simulation study to compare the estimation results based on the standard Gauss-Hermite rule ($n_q = 20$) with those based on the pseudo-adaptive quadrature rule ($n_q = 6$). We generated 500 Monte Carlo samples of size $n = 1,000$ from the joint model (15) - (17) described in Section 3, where the maximum scheduled follow-up time was set as 5 and the average number of longitudinal measurements was about 3 per subject. The overall censoring rate was about 34% (35% for risk 1 and 30% for risk 2). The bias, the standard error (SE), and the estimated standard error (Est. SE) of the parameter estimates are summarized in Table A.1.

[Insert Table A.1 here]

It is seen that the performance of the two methods are similar. Both methods have small bias and their Est. SEs are close to the SEs in most cases.

APPENDIX A.5. Contrast of runtime between three implementations of the variance-covariance matrices of the Empirical Bayes estimates for the linear mixed effects model

We conducted a small simulation study to compare the runtime between the linear calculation algorithm of the variance-covariance matrices of the Empirical Bayes estimates described in Remark 1 of Section 2.2 and the direct implementation (10) over various samples sizes. We also report the runtime of a popular R package **lme4** as a reference. The results are depicted in Figure A.1 below.

[Insert Figure A.1 here]

It is seen from Figure A.1 that applying the simple linear calculation algorithm can yield a speed-up by a factor of 10 to 10,000 when n grows from 10 to 10^5. Our implementation was also significantly faster than the popular R package **lme4** by a factor of 10 to 500 as n grows from 10 to 10^5.

Table A.1: Comparison of bias, standard error (SE), and estimated standard error (Est. SE) between the standard Gauss-Hermite quadrature rule ($n_q = 20$) and the pseudo-adaptive Gauss-Hermite quadrature rule ($n_q = 6$)($n = 1000$)

		Standard			Pseudo-adaptive		
Parameter	True	Bias	SE	Est. SE	Bias	SE	Est. SE
Longitudinal							
Fixed effects							
β_0	10	-0.008	0.043	0.040	0.001	0.043	0.040
β_1	1	-0.003	0.048	0.027	0.002	0.033	0.031
β_2	-1.5	-0.005	0.059	0.058	-0.002	0.058	0.058
σ^2	0.5	0.002	0.018	0.019	-0.001	0.018	0.018
Competing risks							
Fixed effects							
γ_{11}	0.8	-0.025	0.178	0.182	-0.006	0.179	0.182
γ_{12}	-1	0.004	0.126	0.127	-0.005	0.125	0.127
γ_{21}	0.5	0.005	0.193	0.191	-0.005	0.189	0.191
γ_{22}	-1.5	-0.010	0.135	0.138	-0.017	0.134	0.138
Association							
ν_{11}	1	-0.015	0.132	0.129	0.006	0.133	0.128
ν_{12}	0.5	-0.058	0.228	0.202	0.012	0.204	0.209
ν_{21}	0.7	-0.011	0.131	0.131	0.008	0.130	0.131
ν_{22}	0.25	0.022	0.227	0.210	-0.004	0.216	0.215
Random effects							
Σ_{11}	0.5	-0.006	0.041	0.041	-0.001	0.040	0.041
Σ_{22}	0.25	0.001	0.023	0.022	0.001	0.022	0.022
Σ_{12}	0	0.002	0.026	0.024	0.001	0.024	0.024

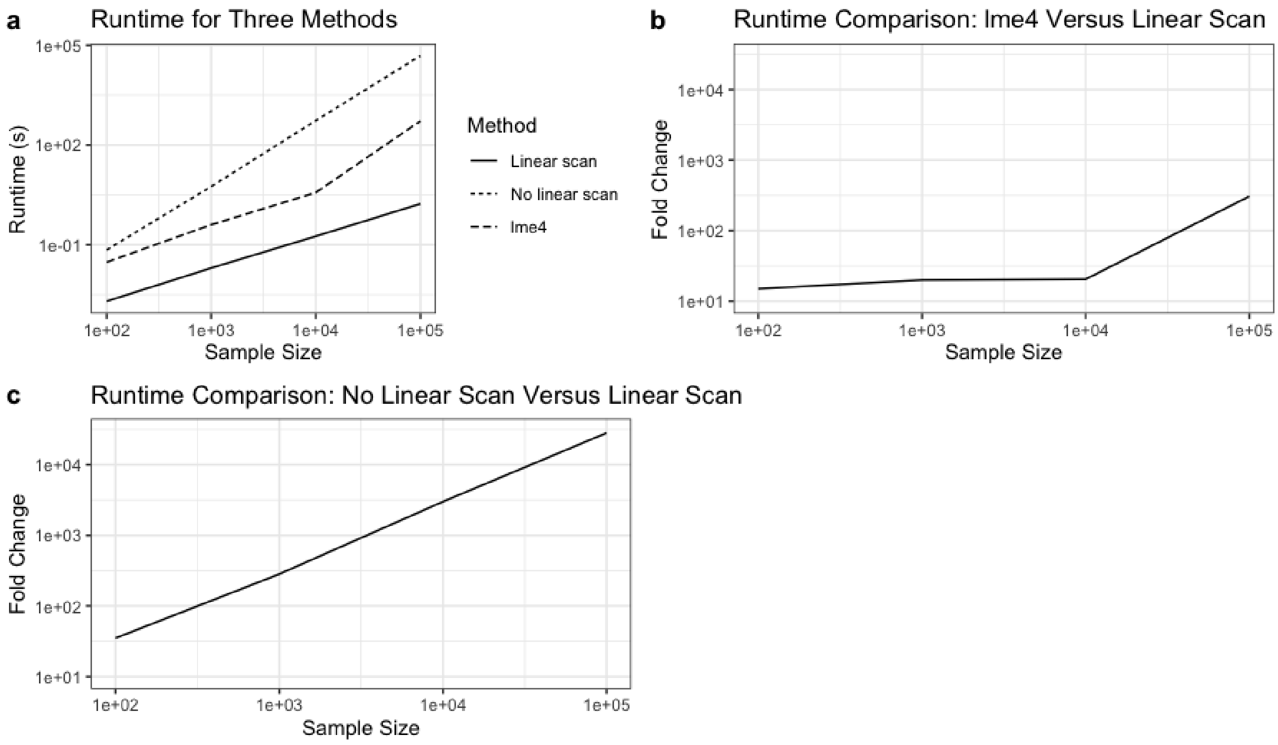

Figure A.1: Runtime (seconds) for three implementations of Empirical Bayes estimates: `lme4`, linear scan, and no linear scan. Fold change is calculated as the ratio of runtime between two methods.

APPENDIX A.6. Parameter and standard error estimation results of different implementations of semi-parametric joint models with competing risks data

Figures 1 and 2 in Section 3 have focused on contrasting the computational efficiency of different implementations for parameter estimation and standard error estimation in terms of the runtime. As an illustration, we ran a small simulation to compare their parameter estimates and estimated standard error based on 100 simulated data sets of size $n = 1{,}000$ from the model (15) - (17). The maximum scheduled follow-up time was set as 5 and the average number of longitudinal measurements was about 3 per subject. The overall censoring rate was about 34% (35% for risk 1 and 30% for risk 2). The estimation results are summarized in Table A.2, where each entry is the average of the parameter and standard error estimates over the 100 simulated data sets. As one would expect, the three different implementations (methods 1-3) yielded almost identical results, whereas `joineR` produced similar estimation results for the longitudinal model, but slightly different results for the competing risks model due to its different latent association structure.

[Insert Table A.2 here]

APPENDIX A.7. Analysis results of the lung health study data

Table A.3 summarizes the parameter estimates with 95% confidence intervals for the joint model (18) and (19) using different joint model packages for the lung health study data.

[Insert Table A.3 here]

It is seen that for the longitudinal sub-model, the parameter estimates obtained from these different packages are almost identical, and that they do not seem to be much influenced by the different latent association parameterization $W_i(t)$ in the survival sub-model. For the survival sub-model, the results are also similar between different packages. The only exception is the association parameter because of the different latent association parameterization $W_i(t)$ in different packages.

It is worth pointing out that both `FastJM` and $\texttt{JMbayes}_b$ fit the same shared random effects model while $\texttt{JMbayes}_b$ assumes a parametric B-spline baseline hazard, and they have

Table A.2: Parameter estimates and standard error (SE) for three implementations of the joint model (1) and (2) and the `joineR` package based on 100 data sets of size $n = 1{,}000$ generated from the model (15) - (17). The details of Methods 1-3 are given in Section 3. Each entry is the average of the parameter and standard error estimates over the 100 simulated data sets.

Parameter	True	Method 1 Estimate (SE)	Method 2 Estimate (SE)	Method 3 Estimate (SE)	`joineR`* Estimate (SE)
Longitudinal					
Fixed effects					
β_0	10	10.00 (0.04)	10.00 (0.04)	10.00 (0.04)	9.94 (0.05)
β_1	1	1.00 (0.03)	1.00 (0.03)	1.00 (0.03)	0.95 (0.02)
β_2	-1.5	-1.51 (0.06)	-1.51 (0.06)	-1.51 (0.06)	-1.52 (0.06)
σ^2	0.5	0.50 (0.02)	0.50 (0.02)	0.50 (0.02)	0.49 (0.02)
Competing risks					
Fixed effects					
γ_{11}	0.8	0.78 (0.18)	0.78 (0.18)	0.78 (0.18)	0.72 (0.20)
γ_{12}	-1	-1.02 (0.13)	-1.02 (0.13)	-1.02 (0.13)	-0.90 (0.11)
γ_{21}	0.5	0.50 (0.19)	0.50 (0.19)	0.50 (0.19)	0.45 (0.19)
γ_{22}	-1.5	-1.52 (0.14)	-1.52 (0.14)	-1.52 (0.14)	-1.44 (0.14)
Association					
ν_{11}	1	1.00 (0.13)	1.00 (0.13)	1.00 (0.13)	N/A
ν_{12}	0.5	0.49 (0.21)	0.49 (0.21)	0.49 (0.21)	N/A
ν_{21}	0.7	0.70 (0.13)	0.70 (0.13)	0.70 (0.13)	N/A
ν_{22}	0.25	0.29 (0.21)	0.29 (0.21)	0.29 (0.21)	N/A
Random effects					
Σ_{11}	0.5	0.50 (0.04)	0.50 (0.04)	0.50 (0.04)	0.52 (0.04)
Σ_{22}	0.25	0.25 (0.02)	0.25 (0.02)	0.25 (0.02)	0.25 (0.02)
Σ_{12}	0	0.00 (0.04)	0.00 (0.02)	0.00 (0.02)	-0.01 (0.03)

*No estimates were available for the association parameters ν because `joineR` uses a different latent association structure.

Table A.3: Parameter estimates using different joint model R packages for the lung health study data

Packages	`FastJM`	`joineR`	`JSM`$_a$	`JSM`$_b$	`JM`$_{a_1}$	`JM`$_{a_2}$	`JM`$_b^*$	`JMbayes`$_a$	`JMbayes`$_b^*$
Parameters (95% CI)									
Longitudinal outcome (FVC%)									
Intercept	5.07	5.07	5.07	5.07	5.07	5.07	-	5.06	5.06
	(4.97, 5.18)	(4.95, 5.17)	(4.97, 5.18)	(4.97, 5.18)	(4.97, 5.18)	(4.97, 5.18)	-	(4.96, 5.17)	(4.96, 5.16)
Time	-0.05	-0.05	-0.05	-0.05	-0.05	-0.05	-	-0.05	-0.04
	(-0.06, -0.05)	(-0.06, -0.05)	(-0.06, -0.05)	(-0.06, -0.05)	(-0.06, -0.05)	(-0.06, -0.05)	-	(-0.07, -0.04)	(-0.06, -0.03)
Age	-0.03	-0.03	-0.03	-0.03	-0.03	-0.03	-	-0.03	-0.03
	(-0.03, -0.03)	(-0.03, -0.03)	(-0.03, -0.03)	(-0.03, -0.03)	(-0.03, -0.03)	(-0.03, -0.03)	-	(-0.03, -0.03)	(-0.03, -0.03)
F10CIGS	-0.003	-0.003	-0.003	-0.003	-0.003	-0.003	-	-0.003	-0.003
	(-0.004, -0.002)	(-0.004, -0.002)	(-0.004, -0.002)	(-0.004, -0.002)	(-0.004, -0.002)	(-0.004, -0.002)	-	(-0.004, -0.002)	(-0.004, -0.002)
Sex									
Female	-	-	-	-	-	-	-	-	-
Male	-0.86	-0.86	-0.86	-0.86	-0.86	-0.86	-	-0.86	-0.86
	(-0.88, -0.83)	(-0.88, -0.84)	(-0.88, -0.83)	(-0.88, -0.83)	(-0.88, -0.83)	(-0.88, -0.83)	-	(-0.88, -0.84)	(-0.88, -0.84)
Logslope	-0.14	-0.14	-0.14	-0.14	-0.14	-0.14	-	-0.13	-0.13
	(-0.15, -0.12)	(-0.15, -0.12)	(-0.15, -0.12)	(-0.15, -0.12)	(-0.15, -0.12)	(-0.15, -0.12)	-	(-0.15, -0.12)	(-0.15, -0.12)
BMI	0.0007	0.0007	0.0007	0.0007	0.0007	0.001	-	0.0004	0.0004
	(-0.002, 0.003)	(-0.002, 0.003)	(-0.002, 0.003)	(-0.002, 0.003)	(-0.002, 0.003)	(-0.002, 0.003)	-	(-0.002, 0.003)	(-0.002, 0.003)
SIP	0.02	0.02	0.02	0.02	0.02	0.02	-	0.02	0.02
	(-0.01, 0.04)	(-0.01, 0.04)	(-0.01, 0.04)	(-0.01, 0.04)	(-0.01, 0.04)	(-0.01, 0.04)	-	(-0.01, 0.04)	(-0.01, 0.04)
SIA	0.03	0.03	0.03	0.03	0.03	0.03	-	0.03	0.03
	(0.01, 0.06)	(0.00, 0.05)	(0.01, 0.06)	(0.01, 0.06)	(0.01, 0.06)	(0.01, 0.06)	-	(0.01, 0.06)	(0.01, 0.06)
SIP:time	0.01	0.01	0.01	0.01	0.01	0.01	-	0.00	0.03
	(0.01, 0.01)	(0.01, 0.01)	(0.01, 0.01)	(0.01, 0.01)	(0.01, 0.01)	(0.01, 0.01)	-	(-0.02, 0.02)	(0.01, 0.05)
SIA:time	0.01	0.01	0.01	0.01	0.01	0.01	-	0.01	0.02
	(0.01, 0.02)	(0.01, 0.02)	(0.01, 0.02)	(0.01, 0.02)	(0.01, 0.02)	(0.01, 0.02)	-	(0.00, 0.02)	(0.01, 0.03)
Error									
σ^2	0.02	0.02	0.02	0.02	0.02	0.02	-	0.02	0.02
	(0.02, 0.02)	(0.02, 0.02)	(0.02, 0.02)	(0.02, 0.02)	(0.01, 0.02)	(0.02, 0.02)	-	(0.02, 0.02)	(0.02, 0.02)
Covariance matrix of b_i									
(Intercept)	0.13	0.13	0.13	0.13	0.13	0.13	-	0.14	0.14
	(0.13, 0.14)	(0.13, 0.14)	(0.13, 0.14)	(0.13, 0.14)	(0.12, 0.15)	(0.11, 0.15)	-	(0.13, 0.14)	(0.13, 0.14)
(Intercept):(time)	0.0012	0.0012	0.0012	0.0012	0.0012	0.001	-	-0.003	-0.0044
	(0.0007, 0.0016)	(0.0011, 0.0012)	(0.0012, 0.0012)	(0.0012, 0.0012)	(0.0011, 0.0012)	(-0.03, 0.032)	-	(-0.0039, -0.0023)	(-0.0090, 0.0002)
(Time)	0.0015	0.0015	0.0015	0.0015	0.0015	0.002	-	0.233	0.234
	(0.0015, 0.0016)	(0.0014, 0.0017)	(0.0015, 0.0016)	(0.0015, 0.0016)	(-0.001, 0.004)	(0, 0.003)	-	(0.225, 0.241)	(0.226, 0.243)
survival outcome (drop out)									
BMI	0.01	0.01	0.02	0.02	0.04	0.02	-	0.01	-0.01
	(-0.01, 0.04)	(-0.01, 0.03)	(0.00, 0.04)	(0.00, 0.04)	(0.02, 0.06)	(0.00, 0.04)	-	(-0.01, 0.03)	(-0.03, 0.02)
SIP	-0.10	-0.10	-0.09	-0.09	-0.06	-0.08	-	-0.07	0.28
	(-0.30, 0.10)	(-0.34, 0.10)	(-0.30, 0.11)	(-0.30, 0.11)	(-0.27, 0.14)	(-0.28, 0.12)	-	(-0.28, 0.10)	(-0.03, 0.59)
SIA	-0.18	-0.18	-0.16	-0.17	-0.14	-0.15	-	-0.15	-0.06
	(-0.38, 0.03)	(-0.39, 0.02)	(-0.37, 0.05)	(-0.38, 0.04)	(-0.35, 0.07)	(-0.36, 0.05)	-	(-0.35, 0.11)	(-0.28, 0.15)
Logslope	0.10	0.09	0.05	0.09	0.16	0.08	-	0.03	0.20
	(-0.02, 0.21)	(-0.07, 0.16)	(-0.07, 0.17)	(-0.02, 0.21)	(0.04, 0.27)	(-0.04, 0.20)	-	(-0.08, 0.15)	(0.09, 0.31)
Sex									
Female	-	-	-	-	-	-	-	-	-
Male	-0.11	-0.09	-0.37	-0.08	0.04	-0.28	-	-0.45	-0.11
	(-0.30, 0.09)	(-0.32, 0.11)	(-0.62, -0.11)	(-0.27, 0.10)	(-0.23, 0.30)	(-0.54, -0.02)	-	(-0.71, -0.15)	(-0.37, 0.15)
Age	0.03	0.03	0.02	0.03	0.05	0.03	-	0.01	0.04
	(0.02, 0.04)	(0.01, 0.04)	(0.01, 0.04)	(0.02, 0.04)	(0.04, 0.07)	(0.01, 0.04)	-	(0.00, 0.03)	(0.02, 0.06)
Association*, ν	Shared	Current value of	Current value	Current value of	Current value	Current value	-	Current value	Shared
	Random effect	Latent process		Latent process					Random effect
	-0.43 (Intercept)	-0.37	-0.33	-0.37	0.11	-0.23	-	-0.45	-0.60 (Intercept)
	(-0.69, -0.17)	(-0.64, -0.17)	(-0.55, -0.11)	(-0.59, -0.15)	(-0.12, 0.34)	(-0.45, -0.01)	-	(-0.69, -0.15)	(-1.11, -0.28)
	0.93 (Time)								14.86 (Time)
	(-3.56, 5.42)								(13.19, 16.42)

*Association parameters that capture the latent relationship between two sub-models have different interpretations among different packages and cannot be compared directly.

*`JM`$_b$ failed to converge and thus no parameter estimates were provided.

*Some parameter estimates obtained from `JMbayes`$_b$ might not converge. Diagnostic plots of the posterior parameter estimates are provided in supplementary materials (See Figure A.2).

produced similar results for all parameters except for the association parameter of random slope of time. A diagnostic analysis for the posterior estimates of the association parameter (Figure A.2) reveals that `JMbayes`$_b$ suffered a convergence issue for this parameter, which explains the discrepancy between the `FastJM` and `JMbayes`$_b$ results.

[Insert Figure A.2 here]

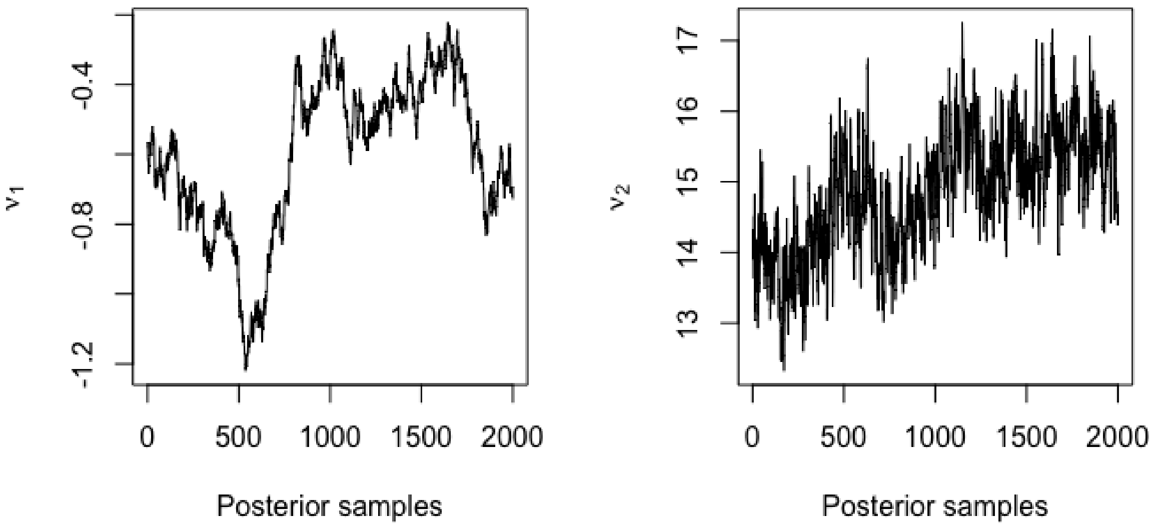

Figure A.2: Diagnostic plot of posterior estimates of the association parameters ν_1 (intercept) and ν_2 (time) based on a Bayesian MCMC sample of 2,000 points obtained from `JMbayes`$_b$ for lung health study data. (MCMC setup: number of iterations = 40,000; number of burn-in = 15,000; number of thinning = 20.)

APPENDIX A.8. Analysis results of the UK-Biobank primary care (UKB-PC) data

Table A.4 shows the analysis results of the `FastJM` and `joineR` packages for the joint model (Model-L) and (Model-PCH) based on two UKB-PC random subsets of sizes n=5,000 and 20,000, as well as the full UKB-PC data (n=193,287).

[Insert Table A.4 here]

Table A.4: Comparisons of parameter estimates for the longitudinal and competing risks survival outcomes for the UK-biobank primary care data between different R packages (n = 5,000, 20,000, 193,287; Type 1 failure = t2d (type 2 diabetes), Type 2 failure = stroke, MI, or all-cause death)

Package	`FastJM`						`joineR`					
Sample size	5,000		20,000		193,287		5,000		20,000		193,287	
Parameter	Estimate	p-value	Estimate	p-value	Estimate	p-value	Estimate	p-value	Estimate	p-value	Estimate	p-value
Longitudinal outcome (SBP)												
Intercept	88.13	<0.0001	88.18	<0.0001	88.48	<0.0001	88.55	<0.0001	88.50	<0.0001	-	-
Age at visit	0.39	<0.0001	0.40	<0.0001	0.40	<0.0001	0.38	<0.0001	0.39	<0.0001	-	-
BMI	0.75	<0.0001	0.74	<0.0001	0.73	<0.0001	0.75	<0.0001	0.75	<0.0001	-	-
Sex												
Female	-	-	-	-	-	-	-	-	-	-	-	-
Male	4.18	<0.0001	3.88	<0.0001	4.01	<0.0001	4.10	<0.0001	3.78	<0.0001	-	-
Ethnicity												
White	-	-	-	-	-	-	-	-	-	-	-	-
Non-white	-2.91	0.0006	-1.49	0.0004	-1.32	<0.0001	-2.97	0.0002	-1.46	0.0001	-	-
σ^2	160.47	<0.0001	160.91	<0.0001	162.65	<0.0001	160.22	<0.0001	160.69	<0.0001	-	-
Covariance matrix of b_i												
(Intercept)	1162.76	<0.0001	1143.11	<0.0001	1088.65	<0.0001	1227.14	<0.0001	1200.78	<0.0001	-	-
(Intercept):(age at visit)	-19.21	<0.0001	-18.92	<0.0001	-18.03	<0.0001	-20.34	<0.0001	-19.94	<0.0001	-	-
(age at visit)	0.34	<0.0001	0.34	<0.0001	0.33	<0.0001	0.36	<0.0001	0.36	<0.0001	-	-
Type 1 Failure												
BMI	0.14	<0.0001	0.13	<0.0001	0.14	<0.0001	0.14	<0.0001	0.13	<0.0001	-	-
Sex												
Female	-	-	-	-	-	-	-	-	-	-	-	-
Male	0.64	<0.0001	0.47	<0.0001	0.48	<0.0001	0.63	<0.0001	0.46	<0.0001	-	-
Ethnicity												
White	-	-	-	-	-	-	-	-	-	-	-	-
Non-white	1.42	<0.0001	1.54	<0.0001	1.28	<0.0001	1.40	<0.0001	1.55	<0.0001	-	-
Association*, ν	0.02	0.0077	0.01	0.0039	0.02	<0.0001	0.01	0.2314	0.0042	0.2439	-	-
	1.17	0.0096	0.52	0.0151	0.97	<0.0001	-	-	-	-	-	-
Type 2 Failure												
BMI	0.05	<0.0001	0.03	<0.0001	0.03	<0.0001	0.04	0.0003	0.03	<0.0001	-	-
Sex												
Female	-	-	-	-	-	-	-	-	-	-	-	-
Male	0.68	<0.0001	0.63	<0.0001	0.62	<0.0001	0.66	<0.0001	0.60	<0.0001	-	-
Ethnicity												
White	-	-	-	-	-	-	-	-	-	-	-	-
Non-white	0.27	0.3671	0.30	0.0258	0.23	<0.0001	0.28	0.3335	0.29	0.0180	-	-
Association*, ν	0.02	0.0217	0.02	<0.0001	0.02	<0.0001	0.02	0.0061	0.02	<0.0001	-	-
	1.42	0.0013	1.51	<0.0001	1.35	<0.0001	-	-	-	-	-	-

*Association parameters that capture the latent relationship between the longitudinal and survival sub-models are different between `FastJM` and `joineR` because of their different latent association structures: `FastJM` uses shared random effects, whereas `joineR` uses the current value of the latent process.

- Fail to produce any result due to computational failure.

It is seen that the analysis results produced by `FastJM` and `joineR` are similar for the longitudinal sub-model for the UKB-PC subset of 5,000 and 20,000 participants. For the survival sub-model, the results are also similar for most parameters except for the association parameters due to the different latent structure $W_i(t)$ between the two packages. It is worth noting that for the full UKB-PC data, `FastJM` was able to finish the analysis in real time (within 1 hour), whereas `joineR` failed to produce any result due to computational failure.
