# Supplementary figures and images for: Efficient Algorithms and Implementation of a Semiparametric Joint Model for Longitudinal and Competing Risk Data: With Applications to Massive Biobank Data
Source: Comput Math Methods Med. 2022 Feb 8;2022:1362913. doi: 10.1155/2022/1362913 (PMC8846996; doi:10.1155/2022/1362913)

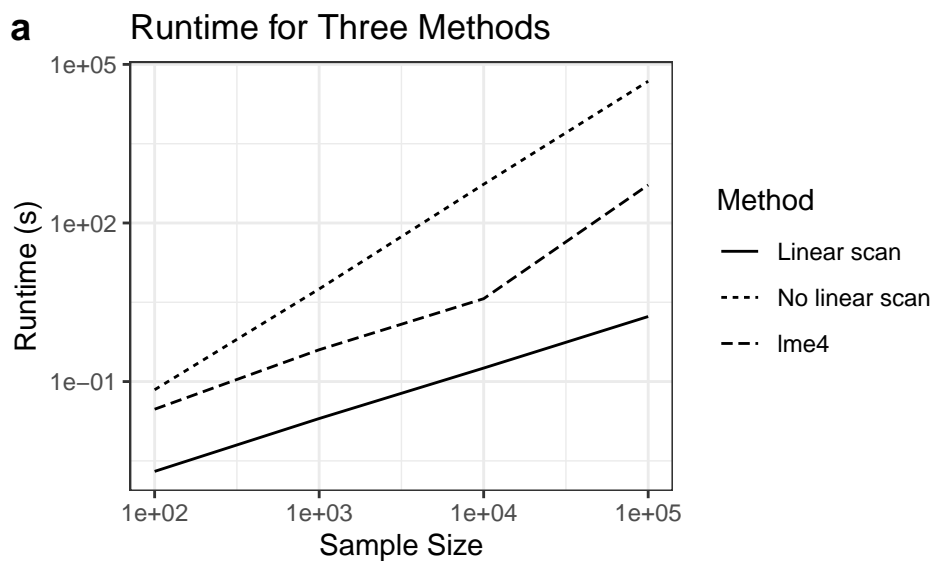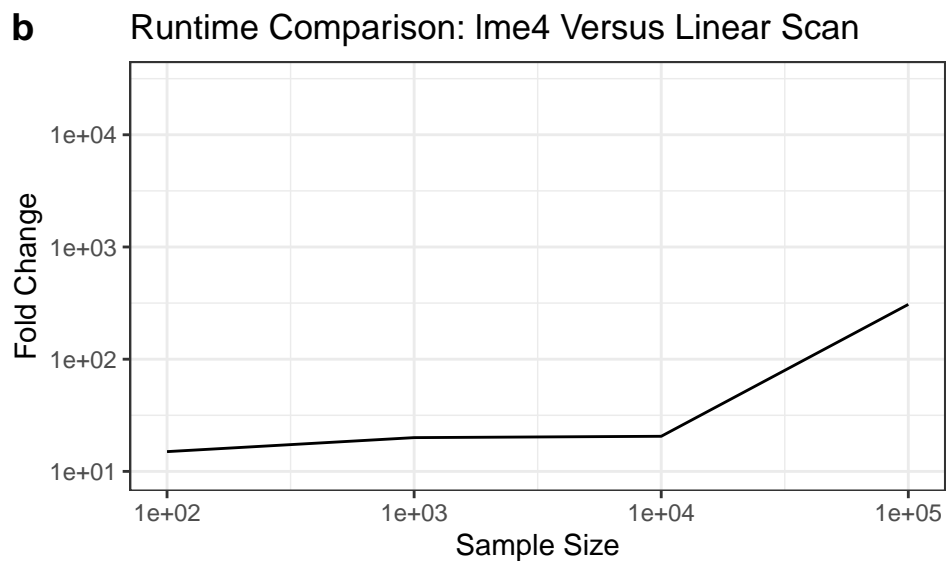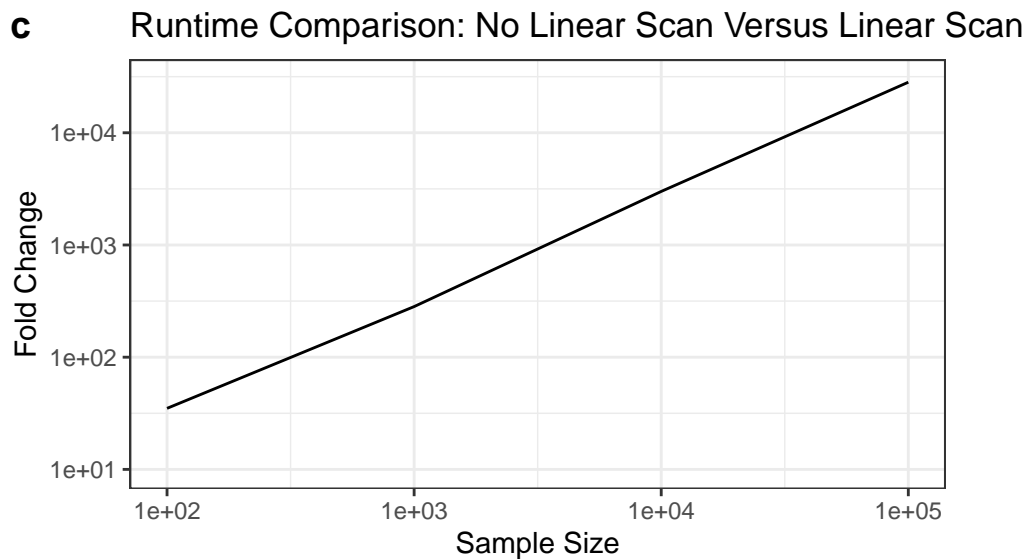

Supplement: Supplementary Materials — Derivations of formulas, additional simulation results, and analysis results of the two real data are provided in the supplementary materials. [file 1362913.f1.zip › Figure A.1 (2).pdf]

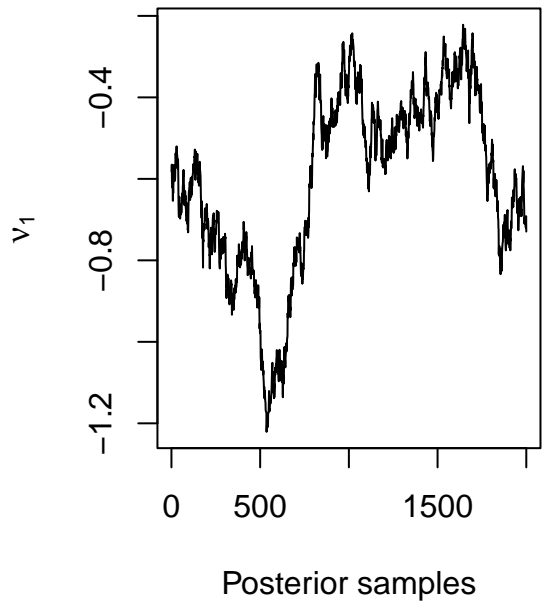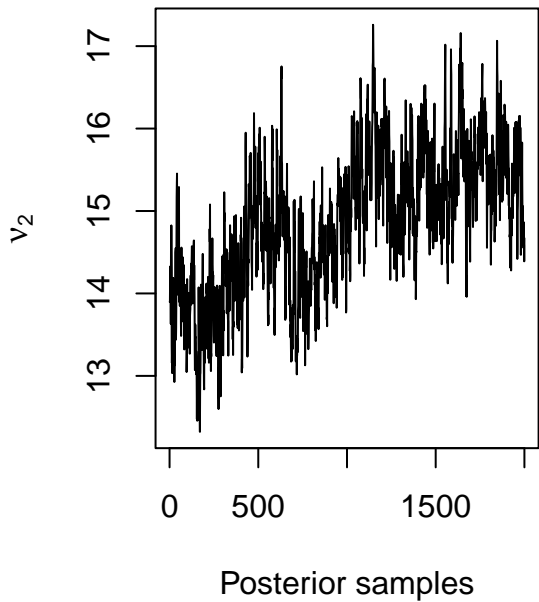

Supplement: Supplementary Materials — Derivations of formulas, additional simulation results, and analysis results of the two real data are provided in the supplementary materials. [file 1362913.f1.zip › FigureA.2.pdf]
